# Une base de connaissance multilingue et dynamique en ligne pour la médecine générale et les soins primaires

**DOI:** 10.11604/pamj.2019.32.66.15952

**Published:** 2019-02-05

**Authors:** Marc Jamoulle, Daniel Knupp Augusto, Miguel Pizzanelli, Ariana de Oliveira Tavares, Melissa Resnick, Julien Grosjean, Stefan Darmoni

**Affiliations:** 1 Département de Médecine Générale, Université de Liège, Belgique; 2 Département d'Information et d'Informatique Médicale, Université de Rouen, France; 3 Société Brésilienne de Médecine de Famille et Communautaire (SBMFC), Curutiba, Brésil; 4 Département de Médecine de Famille, Université de la République (UDELAR), Montevideo, Uruguay; 5 Département de Clinique Générale, Université de Coimbra, Portugal; 6 Medical Librarian, Terminologist, Houston, Texas, United States of America; 7 Département d'Information et d'Informatique Médicale, Université de Rouen, France

**Keywords:** Médecine de famille, soins primaires, classification, base de données terminologiques, Family medicine, primary care, classification, terminology database

## Abstract

**Introduction:**

La classification internationale des soins primaires, deuxième version (CISP-2) alignée sur la classification internationale des maladies dixième révision (CIM-10) est un standard pour le recueil épidémiologique en soins primaires. La CISP-2 convient aussi pour identifier les thèmes cliniques dont discutent les médecins de famille. Les domaines contextuels de connaissances spécifiques à la médecine de famille et aux soins primaires comme les structures, la gestion, les catégories de patients, les méthodes de recherche, les aspects éthiques ou environnementaux ne sont pas standardisés et reflètent le plus souvent des vues d'experts.

**Méthodes:**

Une méthode de recherche qualitative, appliquée à l'analyse de plusieurs congrès de médecine de famille a permis d'identifier, en plus des items cliniques, un ensemble de concepts contextuels abordés par les médecins de famille lors de leurs échanges pendant les congrès. Assemblés sous forme hiérarchique, ces concepts ont trouvé leur place au côté de la CISP-2, sous le nom de Q-codes version 2.5, sur le serveur sémantique multilingue multi-terminologique du Département d'Information et d'Informatique médicale (D2IM) de l'Université de Rouen, France. Les deux classifications sont éditées sous le sigle 3CGP pour *Core Content Classification of General Practice*. Ce serveur d'accès libre permet de consulter la CISP-2 en 22 langues et les Q-codes en dix langues

**Résultats:**

Le résultat de l'utilisation conjointe de ces deux classifications, comme descripteurs dans des congrès, pour identifier des concepts dans des textes, ou indexer la littérature grise en médecine de famille et soins primaires est présenté ici dans différentes utilisations pilotes. La validité et la généralisabilité de 3CGP semble bonne au vu des traductions déjà réalisées par des collègues du monde entier et de l'applicabilité de la méthode des deux côtés de l'Atlantique. Toute fois la reproductibilité et la variation inter-codeurs restent encore à tester pour les Q-codes. La question de la maintenance reste posée.

**Conclusion:**

Grâce à cette méthode, on peut mettre en évidence l'extension conceptuelle, la complexité et la dynamique du métier de médecin généraliste et de famille et de médecin de soins primaires.

## Abstract (en)

**Introduction:**

The International Classification of Primary Care, Second version (ICPC-2) aligned with the 10th Revision of the International Classification of Disease (ICD-10) is a standard for primary care epidemiology compendium. ICPC-2 has been also intended to identify the clinical topics in family medicine. Contextual field-specific knowledge in family medicine and primary care such as health structures, management, categories of patients, research methods, ethical or environmental features are not standardized and reflect, more often, the views of experts.

**Methods:**

A qualitative research method, applied to the analysis of several Family Medicine congresses, has helped identify, in addition to clinical items, a spectrum of contextual concepts addressed by family doctors during their exchanges at the congresses. Assembled in a hierarchical manner, these concepts were given expression, together with ICPC-2, under the name of Q-codes Version 2.5, in the multilingual multi-terminology semantic server of the Department of Information and medical informatics (D2Im) at the University of Rouen, France. The two classifications are edited under the acronym 3 CGP for Core Content classification of General Practice. This free access server allows you to consult the ICPC-2 in 22 languages and the Q-codes in ten languages.

**Results:**

The result of the joint use of these two classifications, as descriptors in congress to identify the concepts in texts or index the gray literature for family medicine and primary care is presented here in its various pilot uses. The validity and generalizability of 3CGP appears to be good in the light of the translations already carried out by colleagues around the world and of the applicability of the method in the two sides of the Atlantic. However the reproducibility and the inter-coder variations still remain to be tested for Q-codes. Maintenance remains an issue.

**Conclusion:**

This method highlights the conceptual extension, the complexity and the dynamics of the role of general practitioner and family doctor as well as of primary care physician.

## Introduction

Le contenu de l'enseignement de la médecine générale n'est pas standardisé. Malgré des définitions très élaborées de la médecine de famille, [1] la manière dont la profession est structurée varie énormément entre les recommandations [2, 3] ou les traités [4-8]. Ceci est particulièrement vrai pour les caractéristiques managériales et contextuelles. Ces manuels offrent les vues des personnalités de haut niveau de la profession agissant comme experts. Dans la plupart des facultés francophones, ce sont des médecins spécialistes qui déterminent le programme de formation en médecine et en médecine générale. Peu d'universités appliquent la méthode basée sur les problèmes dont les enseignants sont des médecins généralistes pédagogues [9, 10]. Le médecin de famille est formé dans les universités par d'excellents spécialistes, le plus souvent très orientés vers les maladies rares. Dans son métier pourtant, le médecin de famille voit les maladies les plus fréquentes et est l'interlocuteur quotidien de l'homme souffrant et vieillissant, aux maladies intriquées. Rappelons qu'en termes de problèmes de santé, le Médecin de famille résout à son niveau la plupart des problèmes de santé qui lui sont présentés [11]. Mais à la longue, le temps exprimé par la continuité est un des éléments centraux de la médecine de famille [12], le médecin de famille devient aussi un spécialiste de patients porteurs de maladie rare [13]. Au fil du temps le médecin de famille rencontre les problèmes de santé les plus fréquents. Mais au fur et à mesure des années il aura à accompagner de plus en plus de patients porteurs de maladies rares ou peu fréquentes différente [14]. La probabilité qu'il a de rencontrer telle ou telle maladie peu fréquentes est chaque année minime mais elle est cumulative au cours des ans. Quand il la rencontrera, il n'aura plus que de lointains souvenirs de son enseignement. Il lui faudra alors trouver l'information lui permettant d'accompagner le patient sur la durée. Il lui faudra être plus discriminant et plus rapide que ce dernier dans sa quête d'information [15]. Trouver l'information au point de soin n'est pas une matière vraiment enseignée. Dans une enquête récente Brassil *et al.* [16] montrent que la plupart des médecins interrogés n'ont aucune formation formelle en matière de recherche dans les bases de données, ni ne sont au courant de l'existence de nombreuses ressources numériques. Notre propos est d'identifier, en interrogeant les acteurs de terrain, les domaines de compétence qui les préoccupent et de leur fournir des instruments documentaires leur permettant de répondre à leurs besoins.

## Méthodes

Une grille de la connaissance clinique spécifique à la médecine de famille est déjà disponible dans la classification internationale des soins primaires, deuxième édition (CISP-2) celle-ci, alignée sur la classification internationale des maladies dixième révision (CIM-10) est un standard pour le recueil épidémiologique en Soins Primaires [11, 17, 18]. Il s'agit là d'un outil de gestion du champ de connaissance clinique, soit de la liste des problèmes de santé les plus fréquents ou les plus importants ainsi que des procédures mis en œuvre au premier niveau de soin. Les médecins de famille ont développé un autre savoir, non clinique celui-là, que nous appelons contextuel, et qui leur permet de traiter de l'organisation des soins, des relations aux patients, de la recherche ou de l'éthique. Que ce soit au niveau régional, national ou international, les médecins de famille sont aussi des grands producteurs de communications lors de congres. Rien que dans les congrès annuels organisés par l'Organisation mondiale des médecins de famille (WONCA) sur chaque continent, on compte déjà plusieurs milliers d'abstracts. En analysant ces communications, on a pu faire remonter du terrain la connaissance nécessaire à l'exercice du métier et établir une grille du savoir contextuel nécessaire à son exercice. Une étude qualitative, conduite sur plus de deux milles résumés de communications de médecins généralistes lors de congrès, a permis la mise en évidence de champs de savoir spécifique à la médecine de famille. Une liste de 187 concepts a été élaborée dans une classification hiérarchique [19, 20]. Ces concepts sont organisés en 8 domaines qui traitent de la gestion de la connaissance et ce y compris l'enseignement, des questions d'organisations, d'éthique, d'environnement, de la recherche ou des relations aux patients. Un domaine supplémentaire (QO autre) est réservé aux codages de situations imprécises ou aux concepts qui apparaissent de novo et devraient être inclus dans une version ultérieure. L'ensemble est proposé sous le nom de Q-Codes version 2.5. La lettre Q a été choisie car elle était disponible dans la CISP-2. Les deux classifications, l'une clinique, la CISP-2, l'autre contextuelle, les Q-codes, sont éditées conjointement sous le sigle 3CGP pour Core Content Classification of General Practice sur le serveur multilingue multi-terminologique www.hetop.eu/3CGP/fr.

## Résultats

**Construction d'une base de données des thèmes discutés par les acteurs de terrain:** on montre ici les domaines d'application et les résultats déjà acquis par l'indexation de travaux de médecins de famille au moyen de 3CGP.

**3CGP sur le serveur terminologique HeTOP:** le serveur HeTOP édité par le département d'information et d'informatique médicale (D2IM) de l'université de Rouen, France [21] édite 70 terminologies dont la CISP-2 et les Q-codes. Le projet est détaillé sur http://3cgp.docpatient.net/. Les rubriques de la CISP-2 y sont disponibles en 22 langues et les Q-codes en 10 langues. Outre le libellé, chacun des 187 Q-codes comprend une définition, une description de contenu conceptuel, une sélection d'articles en accès libre explicitant le concept, un lien vers la base de données sémantique Babelnet.org et un lien à la base Dbpedia, mère de Wikipédia. En outre, les Q-codes sont mis en relation avec les *Medical Subject Headings* (MeSH) de la *National Library of Medicine.* La question des alignements aux MeSH les plus appropriés a fait l'objet d'une étude approfondie décrite par ailleurs [19]. Ces alignements permettent un lien automatisé à la banque de données bibliographique de langue française LISSA (un million d'entrées) [22] et à PubMed. Les Q-codes et la CISP-2, préparés pour le Web sémantique, sont exportables en tableur Excel et dans le Web Ontology Language (OWL) ce qui permet leur usage dans l'internet sémantique et en traitement automatique des langues (TAL) comme dans l'extracteur automatique de concepts à partir de textes ECTMV3 [23].

**Figure 1:**
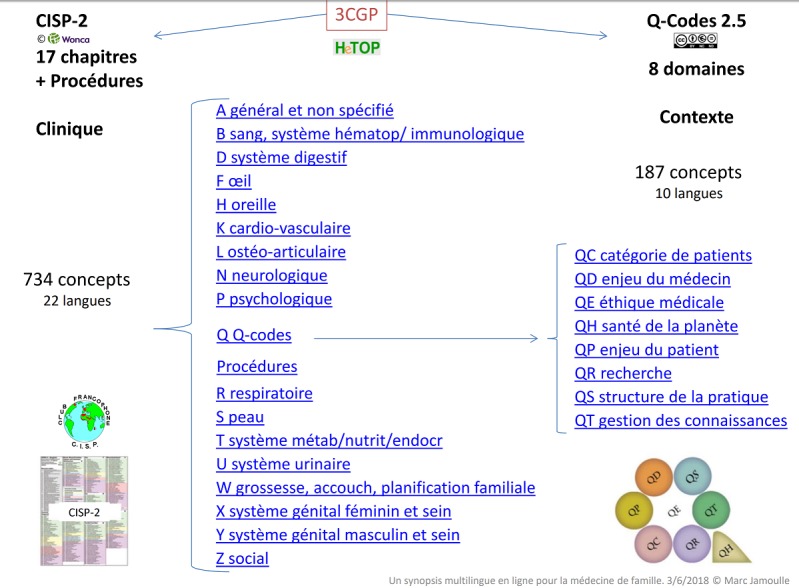
Les 17 chapitres de la CISP-2, la CISP-2 Procédures et les 8 domaines des Q-Codes version 2.5 en français

**La CISP-2 et la base de Q-codes version 2.5 sur le serveur HeTOP:** chacune des 784 rubriques de la CISP-2 et les 187 Q-codes disposent d'une adresse internet spécifique appelée identifiant uniforme de ressource, (URI - Uniforme Resource Identifier) qui permet la gestion informatisée de l'information. Dans la liste des domaines présentée à la Figure 1 les entrées sont interactives et permettent au lecteur d'explorer les possibilités de la base de données. Il y a un URI par item et par langue choisie. Des médecins généralistes de nombreux pays se sont montrés intéressés et volontaires pour traduire et les Q-codes sont disponibles en français, anglais, espagnol, portugais, néerlandais, turc, kartuli (Géorgie), ukrainien, coréen et vietnamien. D'autres traductions sont en cours.

**Publication de terminologies multilingues de la médecine de famille:** une publication imprimée a été réalisée reprenant exclusivement les Q-codes et leur définitions (appelées aussi note de contenu) ainsi qu'un historique et un mode d'emploi. Ces ouvrages constituent un dictionnaire des aspects contextuels de la médecine de famille et des soins primaires. Ils sont disponibles en français, anglais, néerlandais, espagnol, portugais et vietnamien sur le site http://3cgp.docpatient.net/the-book et sous forme de livres [24].

**Utilisation des hyperliens dans des textes d'enseignement:** à titre d'exemple d'utilisation, on lie ici les concepts d'un texte concernant la médecine générale. Il suffira au lecteur désireux d'approfondir la signification des concepts soulignés de cliquer sur le lien: « On sait que la médecine de famille et les soins de santé primaires (SSP) partagent les concepts de continuité, globalité et accessibilité des soins. Toutefois, on a montré que les SSP concernent la structure des pratiques tandis que la médecine de famille concerne la gestion d'un métier bien qu'ils prônent tous deux la prise de décision partagée dans la relation médecin patient » [25].

**Support de recherche bibliographique:** le lecteur trouvera sur www.hetop.eu/3CGP/fr de quoi alimenter sa connaissance en même temps qu'un accès à un filtre bibliographique sur l'item choisi pour tous les Q-codes et pour 20% des codes CISP-2. La façon d'utiliser ce support pour la recherche bibliographique est décrite par ailleurs [26]. A titre d'exemple on peut consulter le sous-domaine QD4 Prévention. On aura ainsi accès aux quatre formes de prévention, à leur illustration et à un filtre spécifique pour chaque item présenté. La question difficile des descripteurs MeSH concernant la prévention secondaire et la prévention quaternaire (QD44) a été traitée par ailleurs en détail [19]. Le filtre sur la Prévention quaternaire a été créé dans HeTOP en texte libre (TW"Text word" dans PubMed) en raison de l'absence de descripteur MeSH pour ce concept. Le filtre sur QD44 génère la stratégie de recherche suivante; *("no harm"[TW] OR “quaternary prevention”[TW] OR “prevention of medicine”[TW])* Cette stratégie de recherche dégage 1671 citations sur PubMed en date du 14 mars 2018. Si on croise cette stratégie avec celle dégagée par le Q-code QS41 médecine de famille, on réalise un deuxième filtre, spécifique à la médecine de famille qui délivre 58 citations. Si on croise avec le Q-codes QS1 Soins de Santé Primaires on obtient 29 citations. Ce croisement de filtres est aisé à réaliser en utilisant le module *My NCBI* qui permet à l'utilisateur d'enregistrer ses propres filtres [27, 28].

**Indexation de travaux en médecine de famille:** les Q-codes ont été élaborés à la suite de l'analyse qualitative de communications de différents congrès de médecine de famille. Ils ont été utilisés en même temps que la CISP-2 pour indexer 2.300 résumés. Dans leur état actuel, les Q-codes ne représentent que les concepts inclus dans les contenus déjà analysés. On s'attend naturellement à ce que la liste des Q-codes s'allonge avec les nouvelles contributions apportées par les médecins de famille lors de congres. Il est étonnant que, dans les congrès analysés pour la mise au point de l'outil, on ait retrouvé que peu de communications traitant de l'éthique (Q-code QE) ou de questions d'environnement (Q-code QH). Cette tendance se confirme lors des utilisations des Q-codes en application ultérieures. A titre d'exemple on en décrit ci-dessous trois expériences d'utilisation.

**Figure 2:**
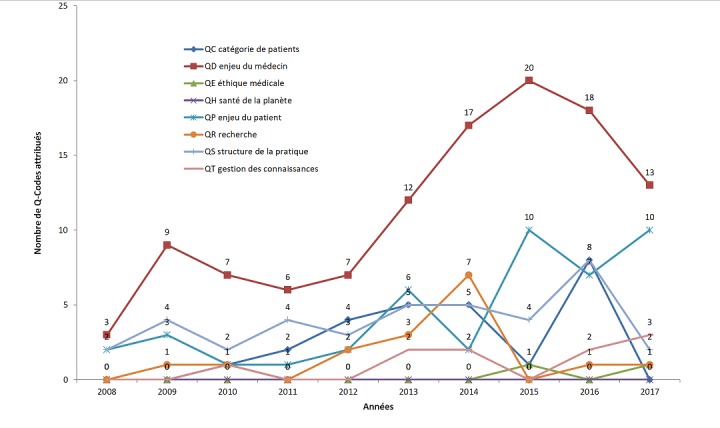
Évolution temporelle de l'application des Q-codes dans les travaux finaux de la 6ème année 370 de médecine 2008-2017

**Université de Coimbra, Portugal, faculté de médecine, travaux de fin d'étude:** trois universités belges ont décidé d'utiliser 3CGP comme descripteur des travaux de fin d'étude (TFE) des médecins de famille en fin de formation. Un guide d'indexation (voir www.mgtfe.be) a été réalisé et incorporé au guide de réalisation d'un TFE [29]. Ce guide et la méthode ont été utilisés par Mme Ariana de Oliveira Tavares, étudiante en médecine en dernière année, pour l'analyse qualitative de 169 travaux de fin d'étude réalisés par des étudiants de la faculté de Coimbra, Portugal, entre 2008 et 2017 [30]. Ces travaux ont été étudiés un à un et codés par la CISP-2 et par les Q-codes. On peut suivre à la Figure 2 l'évolution de la représentation de chaque Q-codes au travers du temps. Les étudiants sont bien sûr attirés par les enjeux du médecin (code QD - courbe rouge) qui rassemblent par exemple la communication, la gestion des problèmes de santé ou la prévention. Les questions éthiques sont peu abordées et les questions d'environnement ne le sont pas du tout. Par contre on voit que les questions relatives aux patients (code QP - courbe bleue)) soit la sécurité, l'accessibilité, les perspectives de vie, etc. attirent de plus en plus les étudiants traduisant peut-être une évolution dans la formation.

**Figure 3:**
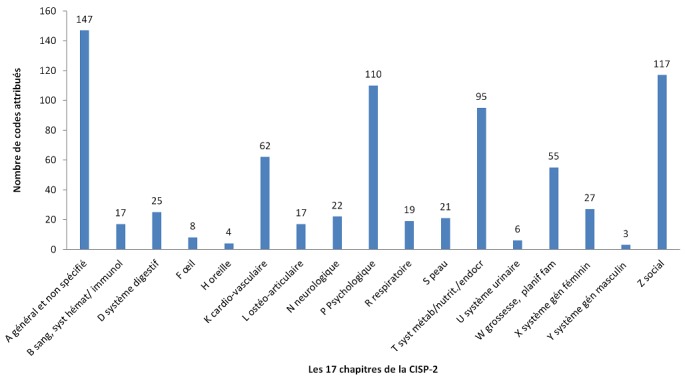
Distribution de 755 codes CISP-2 sur 1746 abstracts acceptés (384 communications orales et 374 1362 posters) SBMFC-Curitiba-2017

**Figure 4:**
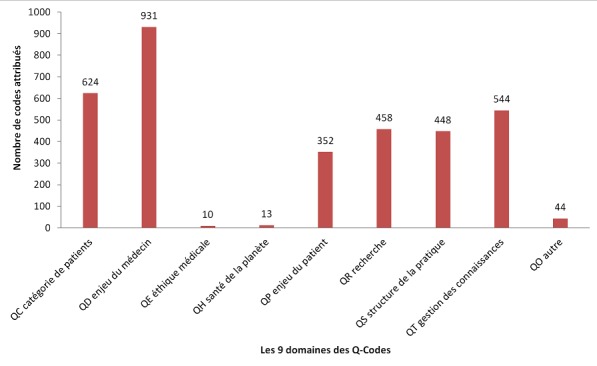
Utilisation des Q-codes comme système d'indexation du Congres SBMFC de Curutiba, 376 Brésil, Novembre 2017

**Utilisation de 3CGP lors du Congrès Brésilien de Médecine de Famille en 2017:** fin 2017, à l'initiative du comité scientifique, le congrès de la Société Brésilienne de médecine familiale et communautaire [28], qui s'est tenu à Curitiba le 7 décembre 2017, a fourni 1746 résumés de contribution encodés par les participants au moment de la soumission en ligne de leur résumé. Les rubriques de 3CGP étaient présentées comme les mots clefs obligatoires. Les Figure 3, Figure 4 montrent les résultats globaux du codage par les déposants. Du point de vue clinique, l'intérêt des participants pour les questions psychologiques (CISP-2 chapitre P) et sociale (CISP-2 chapitre Z), pour la grossesse et la planification familiale (CISP-2 chapitre W) est très tranché. L'importance du chapitre s'explique par de nombreuses communications sur des épisodes préventifs. La distribution constatée est très différente de la distribution habituellement relevée en consultation où les problèmes respiratoires, cardiaques et digestifs sont bien plus nombreux et les problèmes sociaux ne sont quasiment pas relevés. L'importance du chapitre T (nutrition) s'explique par les nombreuses communications consacrées au diabète. On note (Figure 4) que sur les 3.424 Q-codes attribués (soit entre deux et trois par résumé), 931 contributions ont portés sur les enjeux du médecin (QD), 624 sur les catégories de patients (QC), 352 sur les enjeux du patient (QP), 458 sur la recherche (QR), 544 sur l'enseignement (QT). Le code QO utilisés 44 fois indiquent que le déposant n'a pas trouvé le code correspondant à son thème. Une étude minutieuse a permis de réattribuer chaque thème. Ce qui est le plus frappant, ce sont le peu de codes concernant l'éthique (QE) et environnement (QH). Ce phénomène se retrouve dans tous les congrès analysés à ce jour et mériterait une investigation complémentaire.

**Figure 5:**
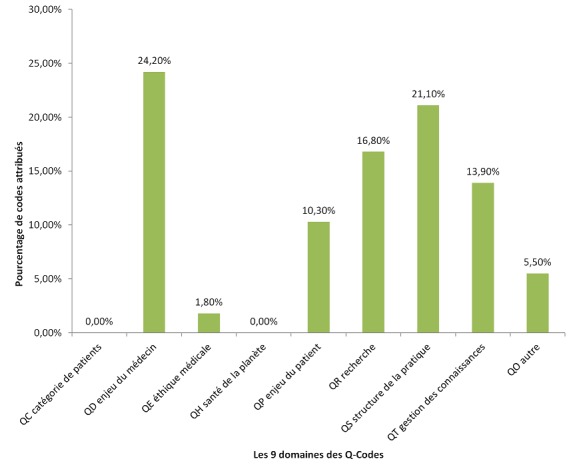
Thèmes contextuels abordés par les jeunes médecins européens, VDGM Porto, Février 2018

**Analyse des thèmes contextuels abordés par le groupe WONCA Vasco de Gama, congrès 2018, Porto:** lors du congrès du groupe WONCA Vasco De Gama 2018 à Porto, Portugal, les jeunes médecins européens ont échangé 97 communications (y compris les *key notes*) codées par un seul observateur (Figure 5). Malgré le petit nombre, en comparaison des 1746 communications auto-codées des médecins brésiliens, on retrouve une distribution similaire des concepts identifiés par les Q-codes version 2.5 et ce malgré la différence de méthodologie de codage [31]. Cette fois-ci il n'y a aucune contribution sur santé et environnement (QH). Sur les 97 communications 7 touchaient à l'éthique avec 1,8% des codes attribués. Il y a 380 codages de caractéristiques contextuelles par 119 Q-codes différents. Les enjeux du médecin (QD) dont la continuité et la globalité représentent 92 codes (24,2%). Les enjeux du patient (QP) comme l'accessibilité, la participation, la sécurité ; 39 codes (10,3%). La structure de la pratique (QS) ; 80 codes (21,1%). On peut souligner que quatre communications sont codées sous QR1 Philosophie des sciences. Les auteurs posent des questions fondamentales sur le positionnement du médecin face à des enjeux idéologiques et politiques et proposent une approche critique face aux défis humains. Le Q-codes QO pour “autre” a cette fois ramené une manne intéressante puisque plusieurs concepts discutés non présents dans la version actuelle ont été retenus pour de nouvelles entrées éventuelles dans la version 2.6 des Q-codes comme; activité physique du patient, test au point de soin, médecine communautaire, nutritionniste, dentiste et orthophoniste.

**Indexation de la littérature grise:** en Uruguay Miguel Pizanelli a créé le projet Not@sLoc@s (notes folles en français, *madnotes* en anglais) dont la moto est *Donnons de la couleur à la littérature grise.* Il s'agit d'un blog Internet où sont rassemblés un ensemble de rapports, travaux d'étudiants, travaux de recherches réalisés pendant la formation des externes et internes de médecine à la faculté de médecine de la république (UDELAR), Montevideo, Uruguay (https://notaslocasmadnotes.wordpress.com/). Il s'agit de littérature grise, soit de travaux non officiellement publiés dont les idées sont intéressantes où qui se réfèrent à de la connaissance contrôlée (au sens de la médecine factuelle (EBM) du terme). Not@sLoc@s est une plate-forme locale conçue tant pour garder trace que soutenir l'échange et la collaboration. Le groupe a un compte Twitter @*RedMadNotes.* La base de données contient toutes les informations concernant un domaine exploré par les étudiants ainsi que les réactions et commentaires. Le travail est classé avec les Q-codes version 2.5 et la CISP-2. Une fonction de recherche permet à un utilisateur de retrouver les articles codés de la même façon.

## Discussion

Bien que les Q-codes ne soient pas exclusivement médicaux, mais aussi managériaux, ils représentent une forme de vocabulaire médical contrôlé, polyvalent, sujet à d'autres ajouts. Les Q-codes sont une liste d'autorité, comprenant une série complète de concepts mutuellement exclusifs et hiérarchisés. Nous avons assemblé des concepts en suivant les directives de Cimino [32]. Soit; *non redondants, partageables, polyvalents, de haute qualité, dans une organisation mono-hiérarchique, identifiés par un ensemble de définitions et liés à des terminologies existantes.* Cet ensemble permet de montrer les connaissances exprimées par les médecins généralistes de plusieurs pays et leurs contributions à la connaissance. Il s'agit donc d'un système de gestion des connaissances en médecine de famille. En complétant la classification clinique CISP-2 par une nouvelle référence contextuelle professionnelle, les Q-codes, on propose de combler une lacune et contribuer à harmonisation de l'enseignement du métier. Conçus comme une ontologie légère multilingue adaptée aux nouvelles technologies de l'Internet, au traitement du langage naturel et au Web sémantique, les Q-codes donnent l'occasion de mettre à jour l'extension, la charge de travail et la productivité de la médecine de famille et de contribuer à en faire une discipline professionnelle digne de respect. Il est important de préciser que les concepts rassemblés dans les Q-codes n'y trouvent leur place que parce qu'ils ont été identifiés dans des résumés de communications analysés. Ceci explique la pauvreté relative des domaines QE (éthique) et QH (environnement) de la classification dans son état actuel. Il ne s'agit donc nullement d'une vue d'experts qui décident d'un contenu mais d'un système de gestion dynamique de la connaissance qui part de la base (bottom-up). L'outil proposé peut faciliter les réseaux d'échange de connaissance, l'organisation de congrès, l'enseignement de la médecine de famille, la gestion bibliographie et montrer l'évolution des pratiques. Le système proposé a été rapidement adopté et traduit par de nombreux collègues de la par le monde. Ceci peut contribuer à lui donner une validité apparente et une généralisibilité, puisqu'il semble subjectivement considéré comme couvrant le concept qu'il est censé mesurer. Mais sa reproductibilité et les variations inter-observatrices n'ont pas pu être testées. Se pose aussi, comme pour toute classification, la question de la maintenance à long terme. Ceci dépendra de l'acceptation par la communauté des médecins de famille du fait qu'il est temps d'identifier et gérer la connaissance qui lui est spécifique d'une façon standardisée et reproductible.

## Conclusion

Le métier de médecin de famille relie la médecine technologique aux humains et les protège des errements de la science grâce à l'expertise du domaine, l'information et la communication. La gestion de l'information, cœur de l'activité, devrait contribuer au développement du métier et à la formation des professionnels des soins primaires.

### Etat des connaissances actuelles sur le sujet

- La formation des acteurs de soins primaires n'est pas standardisée;
- Les tables des matières des *Textbook* de médecine générale sont hétéroclites;
- Le contenu conceptuel des communications des médecins généralistes lors de congrès est peu identifié.

### Contribution de notre étude à la connaissance

- Une analyse qualitative du contenu de congrès de médecine générale permet d'identifier les thèmes discutés et de les organiser dans une nouvelle classification dénommée Q-codes;
- Les Q-codes et la CISP-2 sont proposés en ligne en plusieurs langues sur http://www.hetop.eu/3CGP/Fr;
- Cet ensemble de classifications permet l'analyse de l'activité des médecins généralistes et la gestion de la connaissance en soins primaires.

## Conflits d’intérêts

Les auteurs ne déclarent aucun conflit d'intérêts.
